# Supplementary material for: EnzML: multi-label prediction of enzyme classes using InterPro signatures
Source: BMC Bioinformatics. 2012 Apr 25;13:61. doi: 10.1186/1471-2105-13-61 (PMC3483700; doi:10.1186/1471-2105-13-61)
Supplement: Addtional file 5 — The Java code to format the data files, evaluate and predict. The file enzml_java_code.tar.gz contains the Java code used to format database data to ARFF and XML formats, to execute cross and train-test (jackknife) evaluations and to record evaluation results to database. More information is included in the readme.txt file and the Javadoc files. The code can be used with a MySQL database. To use a different database software, other JDBC drivers might be required. [file 1471-2105-13-61-S5.gz › java_code/utils/doc/uk/ac/ed/inf/utils/stats/PseudoTruncatedPareto.html]

PseudoTruncatedPareto


---


|  |  |  |
| --- | --- | --- |
| **Overview** \| **Package** \| **Class** \| **Use** \| **Tree** \| **Deprecated** \| **Index** \| **Help** | |  |
| PREV CLASS   **NEXT CLASS** | **FRAMES**    **NO FRAMES**     **All Classes** |  |
| SUMMARY: NESTED \| FIELD \| CONSTR \| METHOD | DETAIL: FIELD \| CONSTR \| METHOD |  |


---


## uk.ac.ed.inf.utils.stats Class PseudoTruncatedPareto

```
java.lang.Object
  cern.colt.PersistentObject
      cern.jet.random.AbstractDistribution
          cern.jet.random.AbstractContinousDistribution
              cern.jet.random.Pareto
                  uk.ac.ed.inf.utils.stats.TruncatedPareto
                      uk.ac.ed.inf.utils.stats.PseudoTruncatedPareto
```

**All Implemented Interfaces:**: DoubleFunction, IntFunction, java.io.Serializable, java.lang.Cloneable

---

``` public class PseudoTruncatedPareto extends TruncatedPareto ```

A truncated pareto distribution which stores a big lists of power-law
extracted random numbers. Used to work around the performance problems of the
Pareto distribution.

**Version:**
:   4 Aug 2008

**Author:**
:   Luna De Ferrari luna.deferrari-at-ed.ac.uk

**See Also:**: Serialized Form

---

**Constructor Summary**

`PseudoTruncatedPareto(double scale,
double shape,
int offset,
int truncationThreshold,
int lengthOfPseudoRandomList)`


| **Method Summary** | |
| --- | --- |
| `java.lang.Integer[]` | `getPseudoRandomNumbers()` |
| `int` | `nextPseudoInt()` |

**Methods inherited from class uk.ac.ed.inf.utils.stats.TruncatedPareto**

`getOffset, getPareto, getParetoFrequencies, getTruncationThreshold, nextInt`

**Methods inherited from class cern.jet.random.Pareto**

`cdf, nextDouble, nextDouble, pdf, setState, staticNextDouble, toString`

**Methods inherited from class cern.jet.random.AbstractDistribution**

`apply, apply, clone, makeDefaultGenerator`

**Methods inherited from class java.lang.Object**

`equals, getClass, hashCode, notify, notifyAll, wait, wait, wait`

**Constructor Detail**

### PseudoTruncatedPareto

```
public PseudoTruncatedPareto(double scale,
                             double shape,
                             int offset,
                             int truncationThreshold,
                             int lengthOfPseudoRandomList)
```

**Parameters:**: `scale` - the power law distribution scale: `shape` - the power law distribution shape: `offset` - an offset (all extracted numbers will start from the offset): `truncationThreshold` - a truncation threshold (no extracted number will be above the threshold): `lengthOfPseudoRandomList` - the length of the list of power-law distributed random numbers to be stored


**Method Detail**

### getPseudoRandomNumbers

```
public java.lang.Integer[] getPseudoRandomNumbers()
```

---


### nextPseudoInt

```
public int nextPseudoInt()
```


---


|  |  |  |
| --- | --- | --- |
| **Overview** \| **Package** \| **Class** \| **Use** \| **Tree** \| **Deprecated** \| **Index** \| **Help** | |  |
| PREV CLASS   **NEXT CLASS** | **FRAMES**    **NO FRAMES**     **All Classes** |  |
| SUMMARY: NESTED \| FIELD \| CONSTR \| METHOD | DETAIL: FIELD \| CONSTR \| METHOD |  |


---
